# Molecular Biomarkers and Recent Liquid Biopsy Testing Progress: A Review of the Application of Biosensors for the Diagnosis of Gliomas

**DOI:** 10.3390/molecules28155660

**Published:** 2023-07-26

**Authors:** Yuanbin Wu, Xuning Wang, Meng Zhang, Dongdong Wu

**Affiliations:** 1 Department of Emergency Medicine, The Seventh Medical Center, Chinese PLA General Hospital, Beijing 100700, China; ywu301@outlook.com; 2 Department of General Surgery, The Air Force Hospital of Northern Theater PLA, Shenyang 110042, China; 3 Department of Neurosurgery, The Second Hospital of Southern Theater of Chinese Navy, Sanya 572000, China; 4 Department of Neurosurgery, The First Medical Centre, Chinese PLA General Hospital, Beijing 100853, China

**Keywords:** gliomas, biosensors, molecular recognition and interaction, biomarkers

## Abstract

Gliomas are the most common primary central nervous system tumors, with a high mortality rate. Early and accurate diagnosis of gliomas is critical for successful treatment. Biosensors are significant in the detection of molecular biomarkers because they are simple to use, portable, and capable of real-time analysis. This review discusses several important molecular biomarkers as well as various biosensors designed for glioma diagnosis, such as electrochemical biosensors and optical biosensors. We present our perspectives on the existing challenges and hope that this review can promote the improvement of biosensors.

## 1. Introduction

Glioblastoma (GBM) is the most common primary malignant brain tumor in adults, accounting for about 50% of all gliomas and 15% of all brain tumors [1]. Glioblastoma has the characteristics of infinite proliferation, diffuse infiltration and unclear boundary. It is difficult to completely remove the tumor tissue in surgery, and it is very easy to relapse. The median survival of GBM is less than 15 months, even if surgical resection is performed immediately after diagnosis and combined with radiotherapy, chemotherapy and other comprehensive treatments [2]. Therefore, gliomas are a disease with poor prognosis, and early diagnosis is urgently needed.

At present, the gold standard for diagnosis of a glioma is histopathological examination. However, pathological examination by surgical means, such as biopsy or partial resection, will inevitably cause irreversible damage to normal brain tissue. Therefore, in clinical practice, non-invasive tools such as computed tomography (CT) and enhanced magnetic resonance imaging (MRI) are currently the preferred examination methods for suspected glioma patients, but these two technical means have limited value in evaluating tumor biological activity, invasion ability or potential metastasis [3]. At the same time, the imaging ability of CT for brain and tumor tissue is not as good as that of MRI, and patients need to receive a certain radiation dose; however, MRI has special requirements for the inspection process and field instruments, so it has not been used as a routine item for early screening of glioma in general population physical examination.

Undoubtedly, it would be beneficial for improving the prognosis of patients to find a rapid and effective molecular marker and develop a simple and easy early diagnosis and early warning platform for the tumor. Previous relevant studies have shown that tumor stage and grading determine the prognosis and treatment of patients. For example, the molecular detection of alpha fetoprotein (AFP) enables early detection of liver cancer and improvement of prognosis [4], while the introduction of prostate specific antigen (PSA) screening significantly reduces the mortality of prostate cancer [5]. Glioma is no exception, and its prognosis also depends on early accurate diagnosis and effective individualized treatment.

Liquid biopsy has the advantages of non-invasiveness, high sensitivity and fast detection. It can improve the early diagnosis rate of tumors and is one of the ideal means for early screening of tumors and other diseases in the general population. At present, liquid biopsy techniques for glioma diagnosis mainly include circulating tumor cell (CTC) detection, circulating tumor DNA (ctDNA) detection and exosome detection.

In recent years, biosensors for glioma diagnosis have gained popularity due to their excellent analytical and real-time measurement capabilities. The biological recognition receptors are the key factors affecting the detection effect. They were immobilized on the sensors to interact with the target analytes. In the presence of the target, the biochemical reactions could be converted into electrochemical or optical signals to achieve the purpose of diagnosis. Especially with the combination of advanced materials, the sensitivity of the biosensor will be greatly improved. It is obvious that biosensors provide a particularly promising opportunity for advancing the clinical use of liquid biopsies in patient bedside procedures.

## 2. Glioma Biomarkers

Gliomas are highly heterogeneous tumors. Many molecules can serve as diagnostic, predictive and prognosis biomarkers for a glioma. Here we mainly introduce several important molecular biomarkers which have been researched widely and report some of their recent testing progress.

### 2.1. Major Molecular Biomarkers and Recent Testing Progress in 5 Years

#### 2.1.1. IDH

Isocitrate dehydrogenases (IDH) are central molecular biomarkers of gliomas, which are associated with part of routine histopathological diagnosis, drug sensitivity and prognosis according to the guideline of WHO CNS5 2021 [6]. Although only 12% of patients carry the IDH mutation based on genomic analysis, patients with IDH mutant had better survival. The IDH family includes three isoenzymes: IDH1 in the cytosol and peroxisome, and IDH2 and IDH3 in the mitochondria. IDH is a rate-limiting enzyme for the tricarboxylic acid cycle, which catalyzes the oxidative decarboxylation of isocitric acid to α-KG and CO_2_. IDH family enzymes are activated by ADP, with NAD+ or NADP+ as coenzymes. This reaction catalyzed by IDH is not only an indispensable link in the TCA cycle, but also one of the important sources of intracellular NADPH/NADH and other substances.

**Figure 1:**
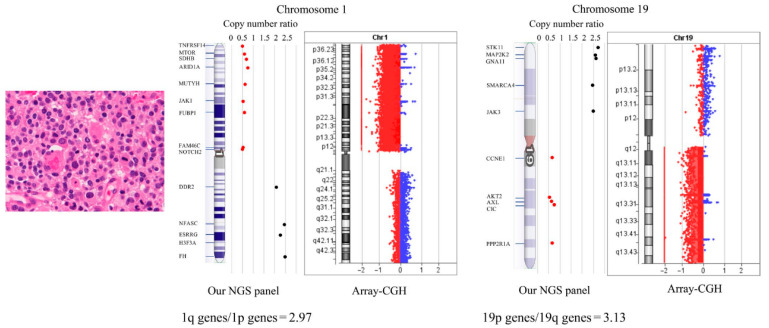
Schematic diagram of a glioma-tailored NGS panel for detecting 1p/19q codeletion and driver gene mutations on a single platform. A patient with anaplastic oligodendroglioma with IDH-mutation and 1p/19q codeletion. For detection of 1p/19q codeletion, they analyzed copy number variations (CNVs) of chromosome 1p loci (9 genes), chromosome 1q loci (5 genes), chromosome 19p loci (5 genes), and chromosome 19q loci (5 genes). To validate 1p/19q codeletion detection, oligodendroglial tumor samples were tested by array-comparative genomic hybridization (CGH). Reprinted with permission from [7].

Next-generation sequencing is clinically used for IDH diagnosis at the present [7], see Figure 1 below. However, many novel approaches have emerged in recent years. An automatic integrated gene detection system (AIGS) has been developed, consisting of cartridges and a device. Reagents for nucleic acid purification and qRT-PCR amplification were placed in the cartridges in advance, as reported by Hao Xue et al. AIGS real-time fluorescence integrates nucleic acid extraction and real-time gene amplification detection, and achieves a fully closed automatic output from samples to genes in the form of a microfluidic card slot. Compared with next-generation sequencing, AIGS has high accuracy in showing results and is a quick and accurate methodology for providing molecular information during surgery [8].

Raman spectroscopy (RS) is a minimally invasive optical technique with great potential for intraoperative diagnosis. Raman analysis showed differences in scattered spectral features of lipids, collagen, DNA and cholesterol/phospholipids. The evidences from 38 unprocessed samples (2073 Raman spectra) showed that RS could distinguish between IDH-MUT and IDH-WT tumors with an accuracy and precision of 87%, with the help of machine learning (RBF-SVM) [9]. With a linear discriminant analysis, the differential mobility spectrometry (DMS) was able to classify IDH mutations in 88 specimens with high classification accuracy, sensitivity and specificity (no less than 80%) [10]. Brown and his colleagues also considered that an ambient ionization strategy, i.e., Desorption Electrospray Ionization, when hyphenated with mass spectrometry (DESI-MS), was able to rapidly differentiate between IDH-MUT and IDH-WT conditions during tumor resection [11,12].

The third-generation nanopore sequencing raised by Oxford Nanopore Technologies shows potential in developing low-cost, high-performance clinical sequencing-based assays with quick turnaround times to provide IDH mutation detections in FFPE tumor tissue [13]. Our team previously reported a strategy for building perovskite quantum dot (PQD)-based biological probes. Given its specific recognition of chlorotoxin for the channels, rapid imaging of glioma cells is successfully performed in 15 min using PQD-based nanocrystals modified with chlorotoxin via charge attraction, providing a nanoplatform for identifying distinct cellular compartments using a different molecular biomarker imaging method [14].

#### 2.1.2. MGMT

MGMT (O6-methylguanine-DNA methyltransferase) is a DNA repair enzyme, located on the long arm of human chromosome 10. Its basic function is to repair damaged guanine nucleotides through transferring methyl groups at the guanine O6 site to cysteine residues [15]. As a consequence of MGMT repairing damaged guanine nucleotide, alkylating agent-induced gene mutations, cell death and tumorigenesis were reduced. Temozolomide (TMZ) is a famous alkylating agent and contributes greatly to the standard treatment for glioma patients. Therefore, the MGMT gene is a factor that contributes to resistance to genetic chemotherapy.

Although the MGMT enzyme is known as an unpleasant protein that reverses and neutralizes the cytotoxic effect of TMZ, epigenetic evidences show that CpG island promoter hypermethylation of the MGMT promoter actually occurs in some glioma cells, then regulates the expression of MGMT genes, which prevents the synthesis of MGMT enzyme [16,17,18]. Due to this inhibition on transcription by DNA methylation, methylation of the promoter region of the MGMT gene can increase cell sensitivity to alkylating agents [19]. Although the optimal cutoff point for MGMT promoter methylation is an issue, MGMT promoter methylation status is definitely a valuable prognostic factor. The gold examination standard for the methylation of MGMT promoter is PCR and a pyrosequencing test [20,21,22].

However, this technique is not widely available in some countries for daily practice. Thus, immunohistochemistry (IHC), an uncomplicated and simpler method, is needed as an alternative examination, but the moderate diagnostic value of IHC can be fully noticed when detecting the real status of MGMT methylation [23,24]. Although this method has several drawbacks, including high interobserver variability and weak correlation between MGMT protein levels and MGMT promoter methylation assessed by other assays, combining a pyrosequencing test and IHC could significantly improve the predictive ability for clinical outcomes [25,26]. The good performance of pyrosequencing tests meets the criteria for application in clinical testing for predictive and prognostication purposes, but the relatively high cost restricts its use in high throughput settings, such as routine tests or large clinical trials [27].

**Figure 2:**
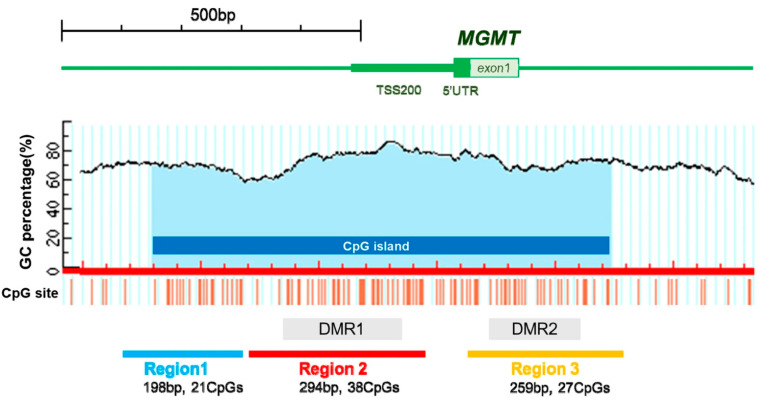
Schematic representation of the MGMT promoter analyzed in the present study assessing MGMT methylation status using high-performance liquid chromatography in newly diagnosed glioblastoma. Reprinted with permission from [33].

Methylation-specific PCR assay (MSP) is simple and low-cost, therefore it is commonly used as a DNA-based molecular diagnostic assay employed for methylation assessment. However, it has some drawbacks, including unstable results and the inability to detect heterogeneous methylation or precisely quantify the exact number of CpG dinucleotides [28,29,30]. Methylation-sensitive high-resolution melting (MH-HRM) is an another method similar to MSP, and the analysis is performed to get methylation quantification [31]. Rosas et al. also clinically validated a novel quantitative MSP assay using double-probe (dp_qMSP) and obtained similar results when compared with conventional MSP [32]. A high-performance liquid chromatography (HPLC) method was recently developed. HPLC employs long PCR products and enables accurate analysis of DNA methylation levels [33], see Figure 2 below. Moreover, a high-density DNA methylation array using the STP-27 algorithm was also proven to be a reliable tool when compared to the classic gold standard assessment by PCR [34]. The Infinium HM-27K and HM-450K BeadChip assay is a high-throughput assay and offers quantification of large epigenomic regions, but it is also expensive and a large sample size is required [35,36].

In addition, Estival et al. evaluated the concordance of MGMT methylation status in paired blood and tissue samples from unresected glioblastomas patients by methylation-specific PCR (MSP) and pyrosequencing. They conclude that the detection of blood cfDNA could be an alternative but the process needs to be improved before it can replace the detection of tumor tissue [37]. Recent advances in MRI-based deep-learning are another trend for noninvasive prediction of MGMT profiling [38,39].

#### 2.1.3. TERT

Telomerase reverse transcriptase (TERT) is one of the important genes encoding the telomerase complex. The human TERT gene is located in the short arm of chromosome 5, containing 16 exons, 15 introns and a promoter region containing 330 base pairs. The TERT enzyme is the most important rate-limiting enzyme and maintains the length of telomeres. Telomerase is strictly regulated in normal somatic cells, but most tumor cells, including gliomas, maintain telomere length by abnormally upregulating TERT expression.

**Figure 3:**
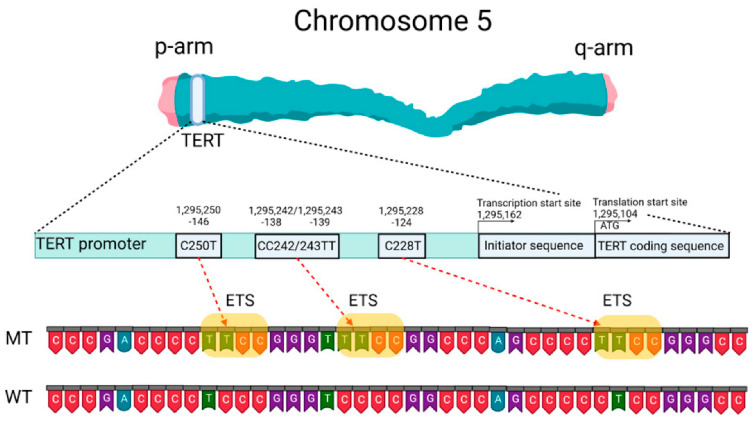
Schematic presentation of TERT gene at chromosome 5p, its promoter structure and two canonical mutations causing gliomagenesis. C > T mutation occurs at one or both positions of the TERTp (−124 and −146 to ATG for C228T and C250T, respectively) in gliomas, which create de novo ETS binding motifs. CC242/243TT is a rare mutation and has not previously been seen in gliomas, although it has been observed in other types of cancer. Reprinted with permission from [40].

TERT mutations occur mainly in the promoter region but rarely in the coding region [40,41], see Figure 3. Gene mutations of TERT promoter (TERTp) cause telomerase reactivation in approximately 90% of solid tumors. Notably, the TERTp region is difficult to amplify because of its high guanine–cytosine content. C228T and C250T are the two recurrent mutations in the TERTp. Approximately 83% of primary GBMs harbor a mutation of C or T in a mutually exclusive manner [42].

The prognostic role of TERT-p mutation in gliomas seems to be ambivalent. The mutation is widely considered as a better prognosis for low-grade gliomas, whereas it tends to be a poor one for GBM. In addition, TERT promoter mutation occurs almost simultaneously with IDH mutations and 1p/19q co-deletion in gliomas [43]. High-throughput sequencing is now used for clinical molecular detection, but the sensitivity depends on the proportion of tumor cells and sequencing depth.

An allele-specific, locked nucleic acid-based quantitative PCR assay was developed to rapidly detect TERTp/IDH mutations with high sensitivity in diffuse gliomas by Bill and his colleagues [44]. One study recommended careful interpretation of targeted NGS data after a comparison of the detection rate of TERT mutations between targeted NGS and Sanger sequencing in 25 IDH-wt glioblastomas [45]. With 100% agreement, iTERT PCR/Sanger sequencing successfully verified the presence of the same mutations in all 103 NGS-verified cases with advanced solid cancers [46]. One study determined the promoter mutation status of BRAF and TERT in 250 papillary thyroid cancers and concluded that ARMS-qPCR was more sensitive than Sanger sequencing [47].

Gliomas have also been successfully analyzed by plasmatic cell-free and tumor DNA according to TERTp mutation detection by droplet digital PCR (ddPCR), which is an ultrasensitive, fast and cost-effective tool [48,49]. TERT promoter mutation in cell-free DNA could also be detected in various cancers other than glimoas, such as hepatocellular and urinary cancer [50,51,52]. At the present, no simple testing modality is available to detect TERT expression in surgical tissues. However, an RNAscope^®^ assay of TERT expression was performed on a Leica Biosystems staining robot with the help of an Hs-TERT-O1 (ACD, 481968) probe. This approach showed a good correlation when compared with RNA-sequencing, implicating this assay as a potentially reliable tool for the evaluation of TERT mRNA expression in formalin-fixed neoplastic tissues [53]. In addition, CT and MRI metrics associated with TERT mutation in gliomas have also been researched for preoperative prediction [54,55,56].

### 2.2. Other Promising Molecular Biomarkers

#### 2.2.1. 1p/19q Co-Deletion

Genetic analysis has revealed that chromosomal translocation can be detected in many carcinomas including gliomas and plays an important role as a driving force in tumorigenesis. t(1;19)(q10;p10) has led to 1q/19q co-deletion, which means a hybrid chromosome containing the 1q and 19p arms is formed [57]. Chromosome 1p/19q co-deletion occurs in 50~70% of WHO grade II and III gliomas. Patients harboring the 1p/19q co-deletion had a better progression-free survival, overall survival and favorable response to chemotherapy [58]. In contrast, 1p and 19q co-deletion are rare mutations in diffuse astrocytic gliomas [59].

#### 2.2.2. ATRX

The ATRX gene, also known as ATP-dependent helicase ATRX, is the pathogenic gene of thalassemia with mental retardation syndrome. The ATRX mutation has been found in several cancers, including glioma, osteosarcoma and pancreatic neuroendocrine tumors. The ATRX protein belongs to the SWI2/SNF2 protein family and acts as a transcription factor, regulating transcription by modifying the local structure of chromatin. ATRX mutations are always associated with IDH mutations. ATRX mutation in glioma disturbed the cell-cycle phase transition, downregulated the expression of Checkpoint Kinase 1 (CHEK1) and even inhibited radio-sensitization [60].

#### 2.2.3. EGFR

EGFR belongs to the ErbB family of tyrosine kinase receptors, which includes HER1 (erbB1, EGFR), HER2 (erbB2, NEU), HER3 (erbB3), and HER4 (erbB4) and is located in the cell surface [61]. These receptors are all transmembrane glycoproteins with molecular weights ranging from 170 to 185 kDa. The EGFR activation involves ligand binding and subsequent receptor dimerization, which is involved in Ras/Raf/MAPK, PI3K/AKT, JAK/STAT or PLC/PKC pathways, affecting a variety of cellular processes, including proliferation, metabolism, apoptosis, cell survival and differentiation [16,59,62].

EGFR amplification is often accompanied by gene rearrangements in GBM. Mutations consist of the deletion of a specific exon or exon fraction. These mutations are classified as EGFRvI (partial deletion at the N-terminus), EGFRvII (deletion of exons 14 and 15), EGFRvIII (deletion of exons 2–7), EGFRvIV (deletion of exons 25–27) and EGFRvV (deletion of exons 25–28). EGFRvIII is one of the most commonly detected gene mutations in GBM. EGFR amplification occurs in approximately 50% of GBM patients, and EGFRvIII rearrangement is concomitant in approximately 50–60% of amplification. EGFRvIII induced a truncated extracellular domain capable of constitutive EGFR activation. The truncated extracellular domain produces a new peptide sequence, resulting in a unique, cell-specific, antibody-reactive EGFRvIII antigen [63,64].

#### 2.2.4. CDKN2A

CDKN2A (Cyclin-dependent kinase inhibitor 2A), which encodes multiple tumor suppressor l (MTS1), is located in 9p21. CDKN2A interacts with CDK4 and CDK6, resulting in inhibiting kinase activity of the complex of cyclin D (CD) and CDK4, reducing the phosphorylation of RB protein [65,66,67]. Those reactions keep the cell in the G phase. CDKN2A deletion had been detected in gliomas, which could serve as a molecular biomarker for prognosis. Furthermore, researchers have revealed the CDKN2A homozygous deletion is a prognostic molecular biomarker for IDH-mutant glioma patients [68].

#### 2.2.5. Exosomes

Extracellular vesicles (EVs) refer to vesicles-like bodies with a double-layer membrane structure that are shed from or secreted by cells; these vesicles are used as “carriers” for cell-to-cell communication and are responsible for the transfer of material between different cells. Exosomes [69] are vesicles with a diameter of 30~150 nm, rich in four transmembrane proteins (such as CD9, CD63, CD81) and other membrane proteins. It has been reported that exosomes exist in all body fluids, including blood, cerebrospinal fluid (CSF), urine, saliva and breast milk. Exosomes contain a variety of substances, including DNA, lncRNAs, miRNAs and proteins based on the cell types and biogenesis. They therefore play an important role in intercellular communication through the transfer of protein and nucleic acid substances [70,71].

Exosomes can participate in physiological and pathological activities such as cancer progression by mediating signal interaction between tumor and stromal cells [72,73]. Compared with normal cells, tumor cells can secrete more exosomes, and tumor derived exosomes carry complex biological information from their mother cells. Research has shown that intracranial tumors can release exocrine bodies in peripheral circulation and cerebrospinal fluid, which provides a new choice for the diagnosis of central nervous system tumors located behind the blood–brain barrier [74,75]. Many molecules in exosomes which could be diagnostic and prognostic biomarkers for gliomas were identified. Therefore, exosomes can be used as GBM biomarkers, and provide certain advantages in early diagnosis and early warning of GBM [76,77].

#### 2.2.6. cfDNA

Cell-free DNA (cfDNA) is a fragmented mixture of DNA composed of DNA molecules released by a variety of tissues. Several existing tumor liquid biopsy studies have demonstrated that the detection of cfDNA mutations is effective in identifying tumor patients. cfDNA can be used to monitor a variety of biological processes, and has been demonstrated in tumors and transplanted organs. cfDNA markers provide an important technical pathway for non-invasive prenatal testing cancer detection (liquid biopsy). In addition to the variation of the sequence of the cfDNA fragment itself, its fragmentation characteristics are also very interesting; The fragmentation of cfDNA presents a multidimensional character that far exceeds expectations; in fact, the fragmentation of cfDNA is not random, but contains information about the organization of its origin [78,79].

#### 2.2.7. ctDNA

ctDNA (circulating tumor DNA) is a part of circulating free DNA, which is a short single- or double-stranded DNA fragment. ctDNA is from somatic DNA which is released into the circulatory system after tumor cell somatic DNA is shed or released into the circulatory system after apoptosis, which is a characteristic tumor biomarker [80,81]. Hence, ctDNA is like the fingerprint left in the blood of tumor cells. It contains the same mutation information as the original tumor, so it can be used to detect tumor related mutations, making it possible to trace the true culprit of cancer and analyze its characteristics with just one tube of blood, thus providing indispensable information for eliminating the murderer. In almost all types of cancer, including gliomas, the signature mutation of ctDNA is detected.

However, its sensitivity limits the clinical application. It is mainly because the content of tumor ctDNA molecules in blood is usually much lower than that of circulating free DNA from non-tumor sources, which makes detection very difficult, especially in the early stage of cancer [82]. For glioma patients, many research results show that compared with other solid tumor patients, the detection rate of plasma ctDNA in glioma patients is lower, making it difficult to effectively promote clinical diagnosis [83,84].

#### 2.2.8. CTCs

Circulating tumor cells (CTCs) originate from tumor tissues and can enter blood and cerebrospinal fluid. Because brain tumors usually do not metastasize through blood due to the blood–brain barrier, less than 40% of patients with glioblastoma have CTCs detected in their peripheral blood. Moreover, because the circulating body fluid of the human body is large and in a constantly updated state, the amount of CTC in the blood is less. Some studies show that it is necessary to detect 105~107 monocytes to detect one CTC, so the extremely low concentration of CTC in the body fluid of GBM patients greatly limits its transformation application [85].

## 3. Available Liquid Biopsy and Their Prospects on Gliomas

Liquid biopsy is the use of body effluents such urine or saliva instead of surgical biopsies, and is a powerful tool for precision clinical practice. Predictive markers serve a crucial role for the evaluation of diagnosis, prognosis and selection of appropriate treatment, especially in gliomas which are difficult to diagnosis and assess progression [86]. Biosensors for a liquid biopsy are made up of three components: the receptor for binding the molecular biomarkers, the transducer for converting the interaction into an alternative signal, and the biosensor analyzer for displaying the result. There are two main categories for liquid biopsy detection in gliomas, electrochemical biosensors and optical or colorimetric biosensors, both of which have high sensitivity and specificity.

### 3.1. Electrochemical Biosensor

An electrochemical biosensor is a kind of transducer that uses electrodes as signal lamps in the system, and active units in biology (such as antigens, antibodies, enzymes, microorganisms or whole cells) as the objects to be tested to identify the original parts, using current, conductance or potential change as the characteristic detection signal. Based on the chemical reaction principle, they are sensors that are converted into electrical signals.

#### 3.1.1. Electrochemical Protein Sensor

An electrochemical protein sensor is a detector that takes a specific protein as a sensitive element and combines the reaction between target and protein as an electrochemical analysis function to output electrical signals. As sensitive recognition elements, proteins mainly include thrombin, metalloproteinase (MMP), horseradish peroxidase and several common heme proteins, such as hemoglobin (Hb), myoglobin and cytochrome [87,88].

#### 3.1.2. Electrochemical Immunosensor

Biosensors based on interactions between antibodies and antigens are called immunosensors, and their recognition elements are biological receptors with a specific binding capacity. They are characterized by easy operation, high sensitivity, low cost, easy integration and small size. Antibodies are one of the most widely used molecules. Because of their high specificity and affinity, they have been used in chromatography, diagnosis, immunoassay and biosensors [89,90].

#### 3.1.3. Electrochemical Nucleic Acid Aptamer Sensor

Nucleic acid aptamer (Apt) is a kind of short chain nucleic acid extracted from a random sequence library of DNA or RNA by Systematic Evolution of Ligands by Exponential enrichment (SELEX). It is characterized by the recognition and binding functions for specific targets, stable properties, easy modification and synthesis, and the ability to combine with toxins, antibiotics, viruses and other target molecules. An electrochemical nucleic acid aptamer sensor refers to the signal generated by the specific reaction with the target with the nucleic acid aptamer as the recognition element so as to achieve qualitative and quantitative detection of the target [91,92].

#### 3.1.4. Electrochemical Microbial Sensor

Electrochemical microbial sensors usually use electrode biofilms as sensing elements, and communicate with the electrode through extracellular electron transfer (EET). Electrode biofilms are usually self-assembled. Electroactive microorganisms can be attached to the electrode as biofilms, because the electrode can be used as a solid electron donor or acceptor for microbial respiration [93,94].

### 3.2. Optical or Colorimetric Biosensor

Optical biosensors display biological recognition events through changes in optical signals to achieve quantitative analysis of chemical and biological information. They have the advantages of high sensitivity, fast speed and multi-channel or high-throughput detection. The application fields include: biological/chemical reaction, early warning of explosives and biochemical poisons, environmental pollution monitoring, food hygiene inspection and analysis and testing of various inorganic substances, organic substances, proteins, enzymes and nucleic acids in biological and medical fields.

#### 3.2.1. Resonance-Based Optical Sensors

With the arrival of the information age, sensor technology has been developed and applied unprecedentedly to meet the growing needs of researchers in the scientific community. Compared with traditional optical sensors, optical sensors based on surface plasmon resonance (SPR) are widely used in medical diagnosis, biochemical sensing and other fields due to their advantages of good integration, high sensitivity and flexible design.

SPR biosensors, optical detection instruments, were developed based on the principle that there is a direct correlation between the change in refractive index of the interface and the degree of attenuation of reflected light formed at the interface by an incident light of a certain wavelength during the recognition and formation of a complex by biomolecules. The optical detection process of surface plasmon resonance biosensors contains three important physical phenomena: the presence of plasma electromagnetic waves on the metal surface; resonance, which occurs when this electromagnetic wave absorbs planar polarized light (p-polarization) parallel to the interface; and the occurrence of plasmon resonance which leads not only to the weakening of the intensity of reflected light, but also can be controlled by the change of the refractive index (dielectric constant) of adsorbates on the metal surface. The characteristics of an SPR biosensor are label-free with a low sample size, real-time dynamic monitoring, high sensitivity, high throughput, and wide application area. SPR biosensors allow real-time, in situ and dynamic observation of interactions among various biomolecules, such as DNA, RNA, polypeptides, proteins, oligoglycans, lipids/sacs, viruses, bacteria, phages and cells, and they have been widely used in drug screening, clinical diagnosis, food detection, environmental control and membrane biology [95,96].

#### 3.2.2. SERS Based Sensors

Surface-enhanced Raman scattering (SERS) is an advanced technique that combines the principles of Raman spectroscopy with nanomaterials to significantly enhance the Raman signal from analytes. SERS-based sensors have emerged as powerful tools for ultrasensitive detection and analysis of various molecules, including biomolecules, environmental pollutants and chemical. SERS-based sensors have been applied in various fields, such as biomedical research, environmental monitoring, food safety and forensic analysis. They have demonstrated great potential for detecting disease biomarkers, monitoring pollutant levels, ensuring the safety of food and water and identifying trace amounts of chemicals or explosives.

In conclusion, SERS-based sensors offer ultrasensitive detection, multiplexing capability, rapid analysis, versatility in sample types and compatibility with other techniques. These features make them promising tools for a wide range of applications requiring highly sensitive and selective detection of target molecules [97,98].

#### 3.2.3. Electrochemiluminescence, Chemiluminescent Biosensor

Electrochemiluminescence (ECL), is a luminescent phenomenon produced by electrochemical reactions and chemiluminescence reactions on or near the electrode surface. By applying a certain voltage to the electrode system modified with electrochemiluminescent emitters, the emitters generate redox products on the electrode surface, then form excited states with the corresponding components in the system through electron transfer. The light radiation emitted when returning to the ground state is ECL. The basis of the ECL analysis method is that the ECL signal has a quantitative relationship with the concentration of analytes. It combines the controllability of electrochemical methods and the high sensitivity of chemiluminescence methods. In recent years, with the development of materials science, new ECL luminogens and signal amplification strategies have promoted the performance of ECL methods. ECL has become an important analytical detection method, widely used in environmental pollutants bioactive molecules, nucleic acids, tumor markers et al. [99,100].

#### 3.2.4. Terahertz Based Biosensors

Supermaterials are artificial electromagnetic materials constructed by the periodic arrangement of metallic unit cells with sub wavelength structures, which can greatly improve the resolution and sensitivity of sensors by amplifying evanescent waves (also known as evanescent waves), enhancing local electromagnetic resonances and achieving sub wavelength resolution, thus providing new research ideas for the design of sensors. This project will foothold the previous experience of the project group in the research and development of terahertz ultramaterials and biomedical instruments, addressing the major application needs in the fields of life science, disease diagnosis, food detection and so on, to develop low-cost, label-free, highly eloquent and highly sensitive terahertz biomedical sensors. Through optimization to improve the cytosolic structural design of the supermaterials, a terahertz supermaterial suitable for sensor applications is obtained in either GaAs or SiO_2_ liner materials. Emphasis is put on the study of the electromagnetic resonance of supermaterials, and the interaction mechanism between supermaterials, terahertz electromagnetic waves and biomaterials such as tumor cells, glucose and urea, to obtain highly sensitive and selective biomedical sensors.

#### 3.2.5. Fluorescence Based Biosensors

As one of the common methods for on-site detection of diseases, fluorescence biosensors have the advantages of low cost, high sensitivity and easy operation. An important part in the construction of fluorescent biosensors is fluorescent molecules, including fluorescent dyes and fluorescent nanomaterials. Fluorescence biosensing detection is mainly based on fluorescence resonance energy transfer (FRET), which generally refers to the phenomenon that if the absorption spectrum of the acceptor has a certain overlap with the emission spectrum of the donor, and the distance of two fluorescent groups is less than 10 nm, the transfer of fluorescence energy from the donor to the acceptor can be observed. Liu et al. developed a switch type fluorescent biosensor for the detection of nucleic acid biomarkers. The current state is fluorescence “off” due to fret and photoinduced electron transfer. When the fluorescent probe specifically binds to the target, the rigid double stranded DNA structure is released from the surface of zirconium porphyrin metal organic framework nanoparticles, leading to a fluorescence “on” state. Ultrafast and ultrasensitive detection of nucleic acid biomarkers within 30 min was achieved by observing the on–off state of fluorescence [101,102].

#### 3.2.6. Colorimetric Biosensors

Colorimetric biosensors have the advantages of simplicity of preparation, ease of readout, low cost and ease of carryover [103]. Colorimetric detection can be achieved by oxidation of peroxidase or nanomaterials with peroxidase-like activity, agglomeration of nanomaterials or addition of dye indicators. Among the available nanomaterials, gold nanoparticles are the most widely used because of their simple preparation, easy modification, strong surface plasmon resonance of the nanoparticles and rapid color change that can be caused by the agglomeration between the particles. The greatest advantage of colorimetry-based biosensors is that the presence of target species in the system can be judged by the naked eye, which is highly suitable for application scenarios in home detection [104].

#### 3.2.7. Near-Infrared Spectroscopy

Near-infrared spectroscopy (NIRS) is a spectroscopic technique that utilizes the near-infrared region of the electromagnetic spectrum, typically ranging from 700 to 2500 nm, to analyze the interaction of light with biological samples [105]. NIRS has been widely used for biomarker detection and analysis, and is a powerful analytical technique that has gained significant attention for its potential applications in biosensing, particularly in the diagnosis of gliomas. This technique utilizes the interaction of infrared radiation with biomolecules to provide valuable information about their chemical composition and structural characteristics.

NIRS offers fast analysis time, allowing for real-time or near real-time monitoring of biomarkers. This rapid analysis makes it suitable for applications requiring quick results, such as point-of-care diagnostics. NIRS can be applied to various biological samples, including blood, tissue, urine and saliva. It can detect a wide range of biomarkers such as glucose, hemoglobin, lipids, proteins and metabolites. This versatility makes NIRS a useful tool in diverse areas, including clinical diagnostics, personalized medicine and biomedical research. However, it is important to note that while NIRS is a valuable tool for biomarker detection, it may have limitations in terms of sensitivity and specificity compared to other analytical techniques. The selection of appropriate wavelengths and calibration models is crucial to ensure accurate and reliable results. Continued research and development in this field have the potential to further enhance its applications in clinical diagnostics and monitoring of disease biomarkers.

NIRS is a versatile and informative technique for biosensor applications, particularly in glioma diagnosis [106]. Its label-free nature, molecular profiling capability, non-destructive analysis, potential for in situ diagnosis and complementarity with other diagnostic tools make it a valuable tool in the fight against gliomas [107]. Continued research and technological advancements will contribute to the successful integration of infrared spectroscopy into biosensor platforms, enabling early detection, precise characterization and improved management of gliomas.

#### 3.2.8. Fiber-Based Biosensors

Fiber-optic biosensor structures mainly have light sources, optical fibers, bio sensitive elements and signal detection systems; among these, the biosensitive elements are the key components of the sensors, and the commonly-used biosensitive elements mainly include antigens, antibodies, enzymes and nucleic acids [108]. The biochemical information generated modulates the physical characteristics of the transmitted light in the fiber, such as light intensity, light amplitude, phase, and so on, by selectively interacting with specific biosensitive elements (i.e., specific binding to antigen or receptor ligand; complementary base pairing of nucleic acid molecules; specificity of enzyme action to substrate). Therefore, this sensor has strong selectivity and very high sensitivity, and it can save the tedious work of separation and purification of the test substances in the analytical process; however, the above-formed complexes or organisms produce similar spectral behavior, which cannot be distinguished by fiber itself, and often indicators or markers, such as enzymes, fluorescent substances, acid-base indicators and iron chelate complexes, are used. Compared with other biosensors, the fiber-optic biosensor exhibits the characteristics of fiber sensing, which is specifically reflected in the following ways: (1) Its anti-interference ability is strong due to the good insulating or shielding effect of the fiber itself, and is not perturbed by the surrounding electromagnetic field. (2) No reference electrode is required, and the probe is miniaturized and convenient to operate. (3) Telemetry is achievable and enables real-time, on-line and dynamic detection. (4) It has rapid response with high sensitivity [109,110].

## 4. Perspectives

In recent years, tumor molecules serving as key prognostic biomarkers have brought hope for many patients with carcinomas. Gliomas are notorious as frustrating tumors, but comprehensive treatments, including traditional surgical resections and chemoradiotherapy, have been administrated for a long time. Moreover, a few prevailing molecular biomarkers showing as strong predictors, such as IDH, MGMT, and TERT, have been researched widely and means of efficiently identifying them are still being developed.

The characteristics of biosensors of high sensitivity, high specificity and low detection limit prove their feasibility in glioma diagnosis. The development trend of biosensors in the future should also pay attention to the following aspects: (1) Biomarkers play an important role in the diagnosis of gliomas. Finding more reliable glioma biomarkers is still an important research direction for glioma diagnosis in the future. The pursuit of accurate and precise molecular detection should never be stopped during this fight against gliomas. (2) Most biological recognition receptors are difficult to synthesize and temperature-sensitive. However, aptamers, as a new molecular recognition element, have the advantages of low cost and ease of synthesis, indicating a major revolution in the field of clinical diagnosis. More robust aptamers targeting glioma biomarkers should be screened, and more aptamer-based biosensors should be constructed. (3) Biosensors should be developed in the direction of high intelligence, integration and miniaturization, providing new opportunities and raising hopes for point-of-care detection. (4) Nanomaterial-based biosensors have the potential to revolutionize the diagnosis of gliomas by enhancing sensitivity, selectivity and portability. Ongoing research efforts aim to overcome challenges and bring these biosensors closer to clinical implementation. The integration of nanomaterials in biosensors for glioma diagnosis represents an exciting avenue for advancing healthcare and improving patient prognosis.

This review emphasizes that a comprehensive examination and analysis of the current state of glioma diagnostic biosensors offers valuable insights into their development direction. By conducting this review, we can identify the advancements made, challenges faced, and future prospects associated with the use of biosensors for glioma diagnosis. By gaining a thorough understanding of the current landscape, we can effectively steer the future development of glioma diagnostic biosensors towards enhancing their sensitivity, selectivity, real-time capabilities and accessibility.

In essence, this review serves as a roadmap for researchers, scientists and developers in the field of biosensors for glioma diagnosis. It highlights the importance of integrating nanomaterials, enhancing sensitivity and selectivity, enabling multi-parameter detection, facilitating real-time monitoring and exploring point-of-care applications. By focusing on these key areas, researchers can work towards developing innovative and improved biosensor technologies that will ultimately contribute to the early detection, accurate diagnosis and effective management of glioma.

By leveraging the insights gleaned from the review, we can collectively strive for breakthroughs and advancements that will significantly impact the field of glioma diagnosis and improve patient prognosis.
